# Ceftolozane/tazobactam versus colistin in the treatment of ventilator-associated pneumonia due to extensively drug-resistant *Pseudomonas aeruginosa*

**DOI:** 10.1038/s41598-022-08307-9

**Published:** 2022-03-15

**Authors:** Bence Mogyoródi, András B. Csékó, Csaba Hermann, János Gál, Zsolt D. Iványi

**Affiliations:** Department of Anaesthesiology and Intensive Therapy, Semmelweis University, 1082 Üllői út 78, Budapest, Hungary

**Keywords:** Infectious diseases, Therapeutics

## Abstract

Resistant strains of *Pseudomonas aeruginosa* are common pathogens in the intensive care unit (ICU), limiting available therapeutic options. We aimed to compare ceftolozane/tazobactam (C/T) with colistimethate sodium (CMS) in the treatment of ventilator-associated pneumonia (VAP) due to extensively drug-resistant (XDR) *Pseudomonas aeruginosa*. A retrospective, observational study was performed at a tertiary care ICU. Clinical and microbiological success rate, 28-day all-cause mortality, and adverse events were compared in patients who received C/T with those treated with systemic CMS. A total of 51 patients were included (18 in the C/T and 33 in the CMS group). Clinical success rates in the C/T and CMS groups were 13 (72.2%) and 10 (30.3%), respectively. On multivariate regression analysis, treatment with C/T was independently associated with clinical success (odds ratio 4.47, 95% CI 1.17–17.08). There was no difference in 28-day all-cause mortality (27.8% and 33.3% in the C/T and CMS group, p = 0.76). Acute kidney injury was more common in patients who received CMS (48.5% vs 11.1%, p = 0.01). In our study, ceftolozane/tazobactam was more efficacious in the treatment of XDR *Pseudomonas aeruginosa* VAP and showed a better safety profile compared to CMS.

## Introduction

*Pseudomonas aeruginosa* is widely recognized as a common pathogen of healthcare-associated infections. A large proportion of these infections are caused by multidrug-resistant organisms, which are declared to pose public health threat by the World Health Organisation (WHO). Data from the 2018 report of the European Antimicrobial Resistance Surveillance Network demonstrated resistance to two or more antimicrobial groups in 19.2% of all *Pseudomonas aeruginosa* isolates^1^. Acquired resistance profiles of *Pseudomonas aeruginosa* were described by a group of international experts^2^: Multidrug-resistant (MDR) was defined as non-susceptible to at least one agent in three or more antimicrobial categories; XDR was defined as non-susceptible to at least one agent in all but 2 or fewer antimicrobial categories. MDR and XDR strains of *Pseudomonas aeruginosa* are particularly frequent in ICU-acquired pneumonia, including hospital-acquired pneumonia (HAP) and ventilator-associated pneumonia (VAP)^3,4^. The observed high frequency of antibiotic resistance limits therapeutic options and possibly results in worse outcomes in the ICU. Detrimental consequences of antimicrobial resistance were also demonstrated in cases of hospital-acquired bloodsteam infections and abdominal infections^5,6^.

In the past two decades, colistimethate sodium (CMS) was revived as a last-resort agent for the treatment of resistant *Pseudomonas aeruginosa* infections. Clinically favourable response following intravenous CMS therapy is reported in a wide range (15% and 84.6%) of cases, depending mostly on the represented patient population, site of infection, and outcome definition^7–11^. It is to be noted that the majority of these studies are observational in nature. A randomized, controlled trial recruiting VAP patients^12^ failed to show noninferiority compared to meropenem, although the study was interrupted due to nephrotoxicity in the CMS group. Nephrotoxicity and a narrow therapeutic window of CMS is also documented by other authors^13,14^. To address these limitations, an international consensus guideline was developed to optimize CMS clinical use^15^.

Ceftolozane/tazobactam (C/T) is a novel type of cephalosporin/beta-lactamase inhibitor, targeting nosocomial infections caused by Gram-negative bacteria. The drug shows excellent activity against extended-spectrum beta-lactamase (ESBL) producing *Enterobacterales* and MDR/XDR strains of *Pseudomonas aeruginosa*^16,17^. In recent years, C/T was EMA approved for treating complicated urinary tract infections (cUTI) and complicated intraabdominal infections (cIAI). Following the results of the ASPECT-NP trial in 2019, it was approved to treat nosocomial pneumonia including HAP and VAP^18,19^. The clinical effectiveness of C/T is reportedly between 54 and 86.8% in the treatment of serious *Pseudomonas aeruginosa* infections^18,20–23^.

More reports of clinical experiences are needed to better define the risk–benefit profile of C/T. To the best of our knowledge, there are no results in the literature regarding C/T therapy in Hungarian tertiary ICUs. Moreover, most studies only focused on the clinical efficacy of C/T itself with lack of a comparator group. The aim of the present work is to report our experience with C/T and to evaluate its efficacy and safety compared to CMS in the treatment of ventilator-associated pneumonia due to extensively drug-resistant *Pseudomonas aeruginosa*.

## Methods

### Study design and setting

This retrospective, observational study was conducted between 01 January 2018 and 31 December 2019 in an academic tertiary care ICU at the Department of Anaesthesiology and Intensive Therapy, Semmelweis University, Budapest, Hungary. The following subjects were eligible for the study: (1) age ≥ 16 years, (2) mechanical ventilation for ≥ 48 h, (3) confirmed XDR *Pseudomonas aeruginosa* lower respiratory tract infection which was not present on admission, and (4) at least 72 h of intravenous targeted antibiotic therapy with either CMS or C/T. Two groups were formed and compared according to the used antibiotic therapy: CMS group and C/T group.

Ethics approval for this study was provided by the Semmelweis University Regional and Institutional Committee of Science and Research Ethics, Budapest, Hungary (Registration number: SE RKEB 58/2020). All procedures performed in studies involving human participants were in accordance with the ethical standards of the institutional and/or national research committee and with the 1964 Helsinki declaration and its later amendments or comparable ethical standards. Since the study was retrospective in nature and data were managed and analyzed anonymously, informed consent was waived by the Semmelweis University Regional and Institutional Committee of Science and Research Ethics, Budapest, Hungary. The study was registered on 20/04/2020 with ClinicalTrials.gov, NCT04352855.

### Data collection

We retrospectively reviewed the clinical records of eligible patients diagnosed with VAP caused by XDR *Pseudomonas aeruginosa*. Data were collected for analysis in Microsoft Excel 2016 (Additional File 1). The following data were recorded: demographics, severity of illness scores, history of smoking and underlying diseases according to the Charlson Comorbidity Index, indication for intensive care (medical or surgical patient), tracheostomy, and the need for intermittent or continuous hemodialysis during intensive care. *Pseudomonas aeruginosa* infection was characterized by the following indices: polymicrobial infection, concomitant bacteremia, type of antibiotics used, days of intravenous antibiotic therapy and adjuvant inhaled antibiotic therapy. Patient outcome data related to clinical and microbiological success, 28-day all-cause mortality and adverse events (AEs) were also collected.

### General definitions

Cases of VAP were identified in patients exposed to invasive mechanical ventilation for at least 48 h according to the 2015 European Centre for Disease Prevention and Control (ECDC) criteria^24^. Diagnostic criteria of VAP are summarized in Table 1.

**Table 1:** Diagnostic criteria of ventilator-associated pneumonia.

Radiological signs	Suggestive image of pneumonia on two or more serial chest X-rays and CT-scans
**AND**	
Systemic signs	At least one of the following
	Fever > 38 °C with no other cause
	Leukopenia (< 4000 white blood cell/mm^3^) or leucocytosis (≥ 12,000 white blood cell/mm^3^)
**AND**	
Respiratory signs	At least one of the following
	New onset of purulent sputum, or change in character of sputum
	Cough, dyspnea, or tachypnea
	Suggestive auscultation
	Worsening gas exchange
**AND**	
Microbiological criteria	At least one of the following
	Positive quantitative culture from broncho-alveolar lavage with a threshold of ≥ 10^4^ colony forming unit
	Positive quantitative culture from endotracheal aspirate with a threshold of ≥ 10^5^ colony forming units

Multidrug resistance in *Pseudomonas aeruginosa* isolates was defined as non-susceptible to at least one agent in ≥ 3 antimicrobial categories. Bacteria were classified as XDR when the isolate was non-susceptible to at least one agent in all but 2 or fewer antimicrobial categories^2^.

The indication, dosage, and duration of the antibiotic therapy was established by the treating physicians together with a consultant infectious diseases specialist. C/T was administered by intravenous infusion over 1 h in a standard dose of 1.5 g every 8 h in 9 patients. After C/T approval to treat nosocomial pneumonia, the dose of 3 g q8h was applied in 9 patients. The dose was adjusted in patients with renal impairment, or in those receiving renal replacement therapy, as indicated in the summary of product characteristics.

Acute kidney injury (AKI) during antibiotic therapy was identified according to the 2012 KDIGO AKI guideline^25^ if one of the following criteria was fulfilled: (1) increase in serum creatinine by ≥ 26.5 µmol/l within 48 h, or (2) increase in serum creatinine ≥ 1.5 times baseline within the previous 7 days, or (3) urine volume < 0.5 ml/kg/h for 6 h.

Severity of acute illness was characterized by the Acute Physiology and Chronic Health Evaluation II (APACHE II) and Sequential Organ Failure Assessment (SOFA) score. APACHE II is a severity of disease classification system based upon physiologic measurements, age and chronic health status^26^. The range of APACHE II score is between 0 and 71 points and an increasing score predicts a higher risk of ICU mortality. A score of 25 represents a predicted mortality around 50% for nonoperative patients. SOFA score assesses the severity of organ dysfunction in respiratory, coagulatory, liver, cardiovascular, renal and neurologic systems^27^. It’s range is 0–24 points. The higher the SOFA score, the higher the likely mortality. A score of 12 represents a predicted mortality around 50%.

Charlson Comorbidity Index (CCI) estimates risk of death from multiple chronic illnesses^28^. Ten-year survival is predicted according to 17 variables classifying comorbid conditions and age. The value of 0 estimates 98%, whereas the value of 7 estimates 0% 10-year survival.

### Patient outcome

A successful clinical outcome was defined as the complete resolution of baseline signs and symptoms of ventilator-associated pneumonia, with no new signs and symptoms of respiratory infection and no need for additional antibacterial therapy. Clinical failure was defined as either the lack of clinical response and/or progression and/or recurrence of signs and symptoms of pneumonia. These definitions were used in order to compare the clinical success rates previously reported in the literature regarding C/T experience^20,22^. Clinical response was evaluated at day 14 or earlier by the end of ICU stay. A recurrent infection was considered within 30 days.

Microbiological outcome was defined as (1) eradication: the absence of isolation of *Pseudomonas aeruginosa*, and (2) persistence: the presence of *Pseudomonas aeruginosa* in lower respiratory tract samples. When repeated microbiological cultures were not available, presumed eradication or presumed persistence was considered in patients with clinical success or clinical failure, respectively.

28-day all-cause mortality after the initiation of targeted antibacterial therapy was also assessed.

AEs were classified according to WHO definitions^29^. An AE was considered serious when the untoward medical occurrence at any dose resulted in death, resulted in prolongation of existing hospital stay or in persistent or significant disability, or was life threatening. The severity of AEs was also assessed.

### Statistical analysis

The number of eligible cases during the study period determined the sample size. Clinical success rate was the primary variable of interest.

Continuous variables were analyzed by the Shapiro–Wilk test for normality. Continuous data were reported as medians and interquartile ranges, and categorical data as absolute numbers of cases and percentages. The Mann–Whitney U test was used for the comparison of continuous variables, while the Chi square test or Fisher’s exact test was used to compare categorical variables. Multivariate analyses were conducted to determine the impact of predictor variables on clinical success rate. Before model building, variance inflation factor (VIF) was calculated to estimate multicollinearity for continuous predictors. The variables were used in a forward stepwise logistic regression model (for enter p < 0.05, for exit p > 0.10). Survival data were analyzed using the Cox proportional hazards model, Kaplan–Meier method, and log-rank test. All tests of statistical significance were two-tailed. A p-value ≤ 0.05 was considered as statistically significant. All analyses were performed in SPSS, version 26.0 (IBM Corp., Armonk, New York, USA).

### Ethics approval and consent to participate

The study was approved by the research ethics board of Semmelweis University (SE RKEB 58/2020). Since the study was retrospective in nature, informed consent was considered unnecessary. All procedures performed in studies involving human participants were in accordance with the ethical standards of the institutional and/or national research committee and with the 1964 Helsinki declaration and its later amendments or comparable ethical standards.

## Results

### Demographics and patient characteristics

**Figure 1:**
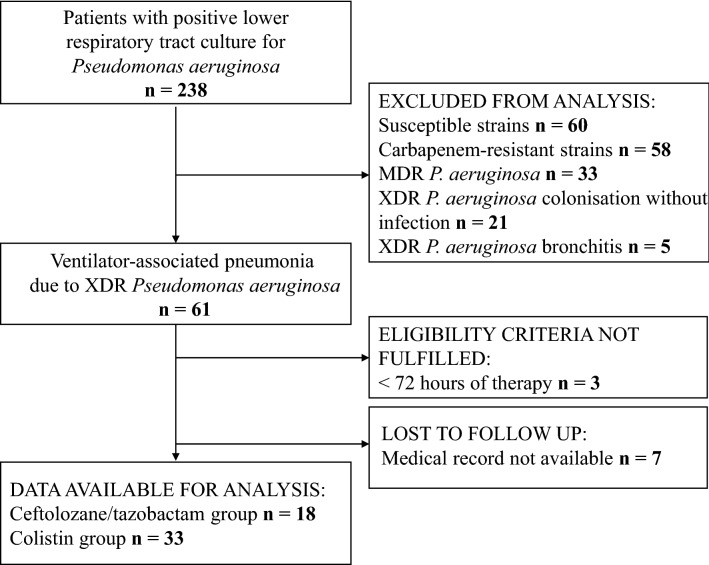
Study flowchart.

A total of 58 patients fulfilled the eligibility criteria and were enrolled. Seven patients were excluded from the study due to incomplete clinical records. Fifty-one subjects were available for analysis. Eighteen patients were assigned to the C/T group and 33 to the CMS group. Study flowchart is shown as Fig. 1.

The median age of the cohort was 66 years (IQR 59–72 years), and 34 (66.7%) were male. The majority of the patients were admitted to the medical ICU (n = 41, 80.4%). The median APACHE II score was 26 (IQR 20–31). The most common comorbidities present included hypertension (n = 33, 64.7%), congestive heart failure (n = 23, 45.1%) and chronic pulmonary disease (n = 15, 29.4%). The median CCI was 4 (IQR 3–6).

Patient characteristics in the C/T and CMS groups are shown in Table 2. Baseline conditions in terms of demographics, severity of acute illness and comorbidities were similar in the two groups without any statistically significant difference.

**Table 2:** Demographics and comorbidities in colistin and ceftolozane/tazobactam treated patient groups.

Variable	CMS group (n = 33)	C/T group (n = 18)	p value
**Demographic factors and severity of illness**			
Age (years)	67 (62–72)	60 (46–72)	0.07^a^
Male sex	23 (69.7%)	11 (61.1%)	0.53^b^
APACHE II	27 (21–34)	25 (20–31)	0.34^a^
SOFA	6 (5–10)	7 (6–9)	0.66^a^
**Preexisting condition**			
CCI	4 (3–6)	3 (1–6)	0.11^a^
Hypertension	23 (69.7%)	10 (55.5%)	0.31^b^
Heart failure	17 (51.5%)	6 (33.3%)	0.21^b^
Cerebrovascular disease	6 (18.1%)	2 (11.1%)	0.69^b^
Peripheral arterial disease	7 (21.2%)	2 (11.1%)	0.46^b^
Lung disease	8 (24.2%)	7 (38.8%)	0.27^b^
Kidney disease	1 (3.0%)	0 (0%)	1.00^b^
Liver disease	2 (6.0%)	1 (5.5%)	1.00^b^
Peptic ulcer disease	4 (12.1%)	4 (22.2%)	0.43^b^
Diabetes mellitus	6 (18.1%)	4 (22.2%)	0.72^b^
Solid-organ tumor	4 (12.1%)	2 (11.1%)	1.00^b^
Leukaemia	3 (9.0%)	0 (0%)	0.54^b^
Corticosteroids	3 (9.0%)	3 (16.6%)	0.65^b^
Other immunosuppressive therapy	2 (6.0%)	2 (11.1%)	0.60^b^
Neutropenia	3 (9.0%)	1 (5.5%)	1.00^b^
Smoking	11 (33.3%)	7 (38.8%)	0.69^b^

*APACHE II* Acute Physiology and Chronic Health Evaluation II score, *CCI* Charlson Comorbidity Index, *CMS* colistimethate sodium, *C/T* ceftolozane/tazobactam, *SOFA* Sequential Organ Failure Assessment score.

^a^Mann–Whitney U test.

^b^χ^2^ test or Fisher’s exact test.

### Treatment characteristics

The median days of antibiotic therapy were longer in the CMS group (9 days, IQR 6–11 days), than for patients who received C/T (7 days, IQR 6–8 days), although the difference did not reach statistical significance (p = 0.18). Polymicrobial infection was detected in 8 patients (24.2%) in the CMS group, and 3 (16.6%) in the C/T group (p = 0.40). In those who received C/T, one was given additional imipenem/cilastatin systemic therapy due to ESBL *Klebsiella pneumoniae* infection. In the remaining 2 cases of polymicrobial infection, combination therapy was not needed to treat *Serratia marcescens* susceptible to C/T. At the time of XDR *Pseudomonas aeruginosa* infection, the median SOFA score was 6 (IQR 5–10) in CMS patients, and 7 points (IQR 6–9 points) in those who received C/T (p = 0.66). Secondary *Pseudomonas aeruginosa* bacteremia was present in 1 (5.5%) patient in the C/T group, and in 3 (9%) patients in the CMS group.

Combination therapy was more frequently used in the CMS group (97% vs 44.4%, p = 0.001) and it consisted of inhaled colistin and systemic beta-lactams (carbapenems and piperacillin/tazobactam). Thirty-two of 33 patients in the CMS group received inhaled colistin in addition to systemic CMS; 3 of them received additional piperacillin/tazobactam and 6 of them were treated with additional carbapenems. In patients who received C/T, the additional antimicrobial agent consisted of inhaled colistin in 6 cases and a systemic beta-lactam in 2 cases. Additional systemic antimicrobial agents were ineffective against the XDR pathogen in all cases.

The median days of mechanical ventilation and days of ICU LOS were 24 days (IQR 13–31 days) and 26 days (IQR 17–33 days) in the CMS group and 20 days [IQR 14–38 days] and 25 days [IQR 19–47 days]) in the C/T group respectively. These differences did not reach statistical significance.

Among patients who received C/T, 9 were given a 1.5 g base dose and 9 received a 3 g base dose (or the renal-adjusted dose). The need for continuous or intermittent renal replacement therapy did not differ in those who received standard or high dose of C/T (4 vs 4 cases). Regarding CRRT in the C/T group, standard dose was used in 2, and high dose in 3 patients.

Treatment characteristics of the C/T and CMS groups are shown in Table 3.

**Table 3:** Treatment characteristics in colistin and ceftolozane/tazobactam treated patient groups.

Variable	CMS group (n = 33)	C/T group (n = 18)	p value
Length of ICU stay (days)	26 (17; 33)	25 (19; 47)	0.49^a^
Duration of mechanical ventilation (days)	24 (13; 31)	20 (14; 38)	0.79^a^
Medical patient	26 (78.8%)	15 (83.3%)	1.00^b^
Surgical patient	7 (21.2%)	3 (16.7%)	1.00^b^
Tracheostomy	19 (57.6%)	9 (50.0%)	0.60^b^
IRRT	7 (21.2%)	5 (27.8%)	0.73^b^
CRRT	7 (21.2%)	5 (27.8%)	0.73^b^
Polymicrobial infection	8 (24.2%)	3 (16.6%)	0.40^b^
*P. aeruginosa* bacteremia	3 (9.0%)	1 (5.5%)	1.00^b^
Days of antibiotic therapy	9 (6; 11)	7 (6; 8)	0.18^a^
Combination therapy	32 (97%)	8 (44.4%)	0.001^b^*

Data are expressed as median (interquartile range) and absolute numbers (percentage).

*CRRT* continuous renal replacement therapy, *CMS* colistimethate sodium, *C/T* ceftolozane/tazobactam, *ICU* intensive care unit, *IRRT* intermittent renal replacement therapy, *P. aeruginosa Pseudomonas aeruginosa*.

^a^Mann–Whitney U test.

^b^χ^2^ test or Fisher’s exact test.

*p < 0.05.

### Outcomes

Clinical success was demonstrated in 13 C/T patients (72.2%) compared with 10 patients (30.3%) who received CMS (OR 5.98, 95% CI 1.67–21.31, p = 0.007). Clinical success rate did not differ in those who received the standard (66.7%, n = 6) or high (77.8%, n = 7) dose of C/T (p = 0.50). Clinical failure in the C/T group was due to death in 4 of 5 patients and persistence of clinical signs and symptoms in one patient. Noteworthy, among the 5 patients who received CRRT in the C/T group, clinical failure was observed in one case where the high dose of C/T was applied.

In multivariate analyses, predictors were included based on results of bivariate comparisons in this study (at a p value ≤ 0.20), and previous publications regarding independent predictors of clinical success or failure (age, Charlson Comorbidity Index, severity of acute illness, polymicrobial infection and continuous renal replacement therapy)^20,30^. The calculation of the variance inflation factors showed no important collinearity among continuous predictors. In forward stepwise model building, only type of antibiotic therapy and age were retained. Age was not significantly associated with clinical success (OR 0.94, 95% CI 0.89–1.01, p = 0.06). In contrast, receiving C/T antibiotic therapy was verified to be an independent predictor for clinical success (OR 4.47, 95% CI 1.17–17.08, p = 0.02).

Proven microbiological eradication was observed in 8 patients (44.4%) in the C/T group, and in 5 patients (15.2%) in the CMS group (OR 4.48, 95% CI 1.18–16.94, p = 0.04). We experienced persistence of XDR *Pseudomonas aeruginosa* in 4 (22.2%) of C/T patients compared with 21 patients (63.6%) who received CMS.

**Figure 2:**
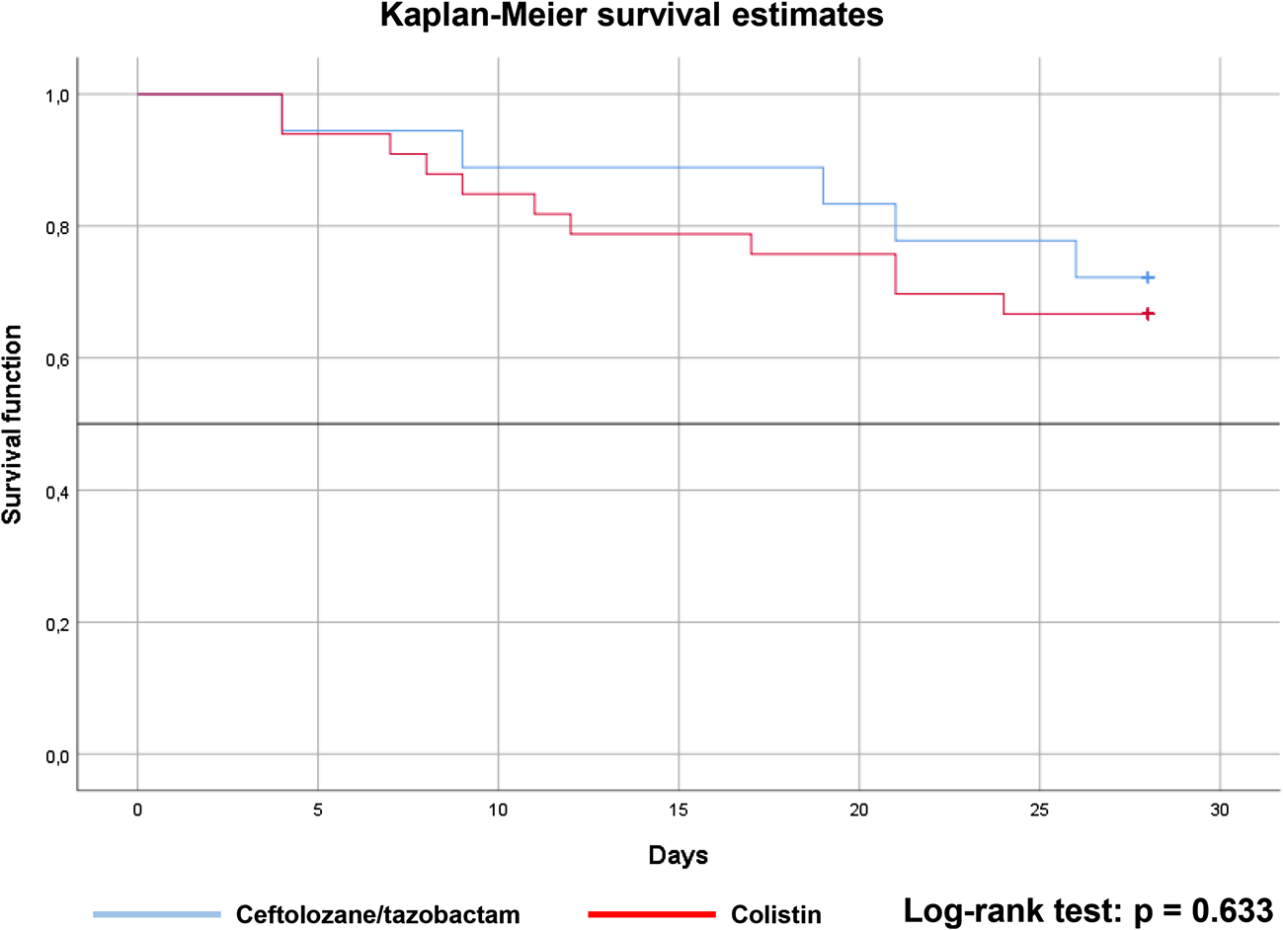
Survival curves for the ceftolozane/tazobactam and colistin groups.

28-day all-cause mortality rate was 5 (27.8%) in the C/T, and 11 (33.3%) in the CMS group (p = 0.76). The results of the univariate Cox regression analysis indicated that targeted antibiotic therapy with either C/T or CMS had no significant effect on survival in the cohort (HR = 0.77, 95% CI 0.26–2.23, p = 0.63). Estimated Kaplan–Meier survival curves showed that patients on C/T treatment tends to have a higher survival rate than those on CMS treatment (Fig. 2). By comparing these survival curves using the log-rank test, no statistical difference was demonstrated (p = 0.63).

### Adverse events

The incidence of at least one adverse event considered by treating physicians was 10 out of 18 patients (55.5%) in the C/T group, and 24 out of 33 patients (72.7%) in the CMS group. The most frequently documented adverse events are shown in Table 4. AEs consisted of gastrointestinal symptoms (*Clostridioides difficile* colitis, diarrhoea, vomiting), asymptomatic increase in liver function tests, rash, atrial fibrillation, and acute kidney injury. Among these, AKI was significantly more common in patients who received CMS: 16 patients (48.5%) experienced AKI in the CMS group and 2 patients (11.1%) in the C/T group (p = 0.01). There were no *Clostridioides difficile* (CDi) colitis observed in the C/T group. In contrast, the rate of CDi colitis was 9.1% in the CMS group, however this difference did not reach statistical significance. Seizures, new-onset neuropathies, and direct Coombs positivity were not observed. All AEs were considered as mild in severity. One patient in the C/T group and 6 patients in the CMS group died during the course of antibiotic therapy and therefore the adverse event is reported as serious. However, no death was considered to be related to antibiotic therapy.

**Table 4:** Adverse events in colistin and ceftolozane/tazobactam treated patient groups.

Most frequent adverse events	CMS group (n = 33)	C/T group (n = 18)	p value
*Clostridioides difficile* colitis	3 (9.1%)	0 (0%)	0.54^a^
Diarrhoea	7 (21.2%)	2 (11.1%)	0.46^a^
Vomiting	10 (30.3%)	2 (11.1%)	0.17^a^
Atrial fibrillation	8 (24.2%)	3 (16.7%)	0.72^a^
Erythema	5 (15.2%)	1 (5.5%)	0.40^a^
Kidney injury	16 (48.5%)	2 (11.1%)	0.01^a^*
**Liver function abnormalities**			
Increased aspartate aminotransferase	6 (18.2%)	4 (22.2%)	0.72^a^
Increased alanine aminotransferase	8 (24.2%)	5 (27.8%)	1.00^a^
Increased γ-glutamyl-transferase	11 (33.3%)	3 (16.7%)	0.32^a^
Increased alkaline phosphatase	8 (24.2%)	1 (5.5%)	0.13^a^

Data are expressed as absolute numbers (percentage).

*CMS* colistimethate sodium; *C/T* ceftolozane/tazobactam.

^a^χ^2^ test or Fisher’s exact test.

*p < 0.05.

## Discussion

The main objective of this study was to report our experiences with ceftolozane/tazobactam and to compare its therapeutic properties to colistimethate sodium. Our results confirmed that ceftolozane/tazobactam is an effective and safe drug to treat ventilator-associated pneumonia due to extensively drug-resistant *Pseudomonas aeruginosa*. The most remarkable observations to emerge from the results were a higher clinical success rate and a lower incidence of AKI and CDi infections associated with C/T, although survival benefit was not demonstrated in this setting.

Even though new antibiotics against multidrug-resistant, Gram-negative bacteria are in development or already approved^31,32^, the treatment of XDR *Pseudomonas aeruginosa* is still challenging for the clinician at the bedside. For this reason, real-world experiences of the efficacy and tolerability of the novel beta-lactam/beta-lactamase inhibitor ceftolozane/tazobactam is of particular interest for the ICU physician. In our cohort, the use of C/T was associated with clinical cure in 72.2%. This observation is relatively consistent with those described in previous studies of C/T therapy (between 73.7 and 86.8%)^20–23^. Most of these studies included patients with both MDR and XDR infections. Among them, the work of Escolà-Vergé et al.^21^ focused on the treatment of XDR *Pseudomonas aeruginosa.* They noted an outstanding clinical success rate of 86.8% at the completion of the treatment. This higher value is likely to be related to the non-critically ill population and the heterogeneous site of infection.

Previous studies showed that higher Simplified Acute Physiology Score II, inadequate source control and continuous renal replacement therapy are associated with clinical failure of C/T treatment^20,21,23^. Severity of acute illness was characterized by APACHE II score in our study and was implied in the multivariate analysis. Results indicate that C/T treatment is an independent predictor of clinical cure (OR 4.47). Interestingly, association between CRRT and clinical failure was not observed, despite of a relatively high proportion of all patients having received this therapeutic modality (23.5%). In a recently published study^33^, the influence of CRRT was investigated in 11 critically ill patients treated with C/T against *Pseudomonas aeruginosa*. They concluded that administration of high dose C/T in extended or continuous infusion and therapeutic drug monitoring should be considered for adequate plasma concentrations. In fact, 3 of 5 patients on CRRT in our study received the high dose of C/T, although clinical success rate did not differ in those who received the standard or high dose of C/T.

In contrast, clinical success was demonstrated in only 30.3% of CMS-treated patients in our study. These findings significantly differ from previous results reported in the literature by Michalopoulos et al.^10^ and Kallel et al.^11^. In the aforementioned studies, intravenous CMS therapy was associated with a favorable clinical response in 69.8% and 75% of patients with ICU-acquired infections caused by CMS-only-susceptible *Pseudomonas aeruginosa* or *Acinetobacter baumannii*. This apparent lack of correlation can be due to the higher incidence of AKI and nephrotoxicity in our CMS group, which was shown to be an independent risk factor of unfavorable infectious outcome^34^. Another possible explanation of this difference is resistance emergence during CMS therapy, although this option was not investigated in this work, since repeated microbiological samples were not collected routinely.

In those where available, proven microbiological cure rate for C/T (44.4%) was relatively lower to those reported previously (53.4% to VAP cases)^22^. Consistently with the results of Gallagher et al. survival was better associated to clinical than microbiological success. Furthermore, since respiratory samples were not collected routinely but when considering a new infection episode, the proven persistence rate of XDR *Pseudomonas aeruginosa* (22.2%) may reflect microbiological results better. On the other hand, clinicians should be aware that pneumonia was also shown as a strong predictor of microbiological failure^22^.

Mortality benefit was not demonstrated with ceftolzane/tazobactam neither in terms of mortality rate (27.8% vs 33.3%), nor by Cox regression analysis (HR 0.77) and the Kaplan–Meier method. More factors may have contributed to the absence of its impact on mortality. First, the effect of antibiotic therapy may be hidden by the severity of acute illness as an important determinant of outcome. In fact, the predicted mortality by the SOFA score calculated at the time of VAP diagnosis was similar in the two groups. Second, since the estimated attributable mortality of VAP is around 10%^35^, a larger study is needed to demonstrate an impact on all-cause mortality. We did not examine the attributable mortality of VAP, because of the potential biases in recording cause of death. This observation is in line with the results of Melsen et al.^36^, who found that the attributable mortality of ventilator-associated pneumonia is lower in medical patients, and that it is mainly caused by prolonged exposure to the risk of dying due to prolonged ICU stay. In our study, the majority of patients were medical patients and the length of hospital stay was nearly identical in the two groups, which could explain the lack of survival benefit.

Ceftolozane/tazobactam had a good safety profile in this study. There were no reported cases of *Clostridioides difficile* colitis, seizures, neuropathy and direct Coombs positivity, despite the fact that half of the C/T patients received the high dose. No new safety signals for ceftolozane/tazobactam were identified.

Antibiotic options for the treatment of resistant Gram-negative bacteria infections include ceftazidime/avibactam. Similarly to C/T, ceftazidime/avibactam is a combination of cephalosporin/beta-lactamase inhibitor and was EMA approved for treating HAP, VAP, cUTI, cIAI and aerobic Gram-negative bacteria infections with limited treatment options^37^. In a review article published by an interdisciplinary group of experts, ceftazidime/avibactam was suggested as a choice of antibiotic therapy to treat carbapenemase-producing *Enterobacteriaceae* nosocomial pneumonia, whereas C/T was suggested to treat *Pseudomonas aeruginosa* nosocomial pneumonia in the ICU^32^. However, in a recently published study by Sader et al., susceptibility rates for C/T and ceftazidime/avibactam were similar in *Pseudomonas aeruginosa* isolates from pneumonia patients^38^. In the largest real-world study reporting *Pseudomonas aeruginosa* infections treated with ceftazidime/avibactam, clinical failure, 30-day mortality and 30-day recurrence occurred in 30.2%, 17.5% and 6.3% of the patients, respectively^39^. In the aforementioned studies a heterogenous population of patients were included focusing not solely on the ICU.

We are aware that a number of limitations might have influenced our results. Possible sources of error are the retrospective nature and the small sample size of the study. We attempted to mitigate this by enrolling all consecutive patients fitting the enrolment criteria. Furthermore, the single-center design of the study may diminish the applicability of our results to other centers. Finally, the diagnostic criteria of VAP, even if they were defined according to the ECDC guideline, remained very subjective^40^, and we may have overestimated the true number of VAP cases. To limit this bias, we included only lower respiratory tract infection episodes microbiologically confirmed and treated with targeted antibiotic therapy. Despite the limitations, our findings support the existing data reported in the only comparative study of ceftolozane/tazobactam and CMS published so far^30^. Nonetheless, our study also has some strengths: the population included only critically ill VAP patients with extensively drug-resistant *Pseudomonas aeruginosa*, and the results were interpreted in contrast to systemic CMS treatment.

## Conclusions

We conclude that ceftolozane/tazobactam should be used preferentially over colistimethate sodium in the treatment of VAP caused by XDR *Pseudomonas aeruginosa*. In addition, ceftolozane/tazobactam is a safe option in avoiding nephrotoxicity during the course of antibiotic therapy. It is important to mention that our conclusions remain to be confirmed in randomized, controlled trials to minimize potential bias.

## Supplementary Information

Supplementary Information.

## Data Availability

All data generated or analyzed during this study are included in this published article [and its Supplementary Information files].

## Abbreviations

AE: Adverse event
AKI: Acute kidney injury
APACHE II: Acute Physiology and Chronic Health Evaluation II score
CCI: Charlson Comorbidity Index
C/T: Ceftolozane/tazobactam
cIAI: Complicated intraabdominal infection
cUTI: Complicated urinary tract infection
ESBL: Extended-spectrum beta-lactamase
HAP: Hospital-acquired pneumonia
ICU: Intensive care unit
MDR: Multidrug-resistant
SOFA: Sequential Organ Failure Assessment score
VAP: Ventilator-associated pneumonia
XDR: Extensively drug-resistant

## References

[1] European Centre for Disease Prevention and Control (2018). Surveillance of antimicrobial resistance in Europe 2018.

[2] Magiorakos AP (2012). Multidrug-resistant, extensively drug-resistant and pandrug-resistant bacteria: An international expert proposal for interim standard definitions for acquired resistance. Clin. Microbiol. Infect..

[3] Koulenti D, Tsigou E, Rello J (2017). Nosocomial pneumonia in 27 ICUs in Europe: Perspectives from the EU-VAP/CAP study. Eur. J. Clin. Microbiol. Infect. Dis..

[4] Fernandez-Barat L (2017). Intensive care unit-acquired pneumonia due to *Pseudomonas aeruginosa* with and without multidrug resistance. J. Infect..

[5] Tabah A (2012). Characteristics and determinants of outcome of hospital-acquired bloodstream infections in intensive care units: The EUROBACT International Cohort Study. Intens. Care Med..

[6] De Waele J (2014). Abdominal infections in the intensive care unit: Characteristics, treatment and determinants of outcome. BMC Infect. Dis..

[7] Montero M (2009). Effectiveness and safety of colistin for the treatment of multidrug-resistant *Pseudomonas aeruginosa* infections. Infection.

[8] Reina R (2005). Safety and efficacy of colistin in Acinetobacter and Pseudomonas infections: A prospective cohort study. Intens. Care Med..

[9] Markou N (2003). Intravenous colistin in the treatment of sepsis from multiresistant Gram-negative bacilli in critically ill patients. Crit. Care.

[10] Michalopoulos AS, Tsiodras S, Rellos K, Mentzelopoulos S, Falagas ME (2005). Colistin treatment in patients with ICU-acquired infections caused by multiresistant Gram-negative bacteria: The renaissance of an old antibiotic. Clin. Microbiol. Infect..

[11] Kallel H (2007). Safety and efficacy of colistin compared with imipenem in the treatment of ventilator-associated pneumonia: A matched case-control study. Intens. Care Med..

[12] Cisneros JM (2009). Colistin versus meropenem in the empirical treatment of ventilator-associated pneumonia (Magic Bullet study): An investigator-driven, open-label, randomized, noninferiority controlled trial. Crit. Care.

[13] Zavascki AP, Nation RL (2017). Nephrotoxicity of polymyxins: Is there any difference between colistimethate and polymyxin B?. Antimicrob. Agents Chemother..

[14] Forrest A (2017). Pharmakokinetic/toxicodynamic analysis of colistin-associated acute kidney injury in critically ill patients. Antimicrob. Agents Chemother..

[15] Tsuji BT (2019). International consensus guidelines for the optimal use of the polymyxins: Endorsed by the American College of Clinical Pharmacy (ACCP), European Society of Clinical Microbiology and Infectious Diseases (ESCMID), Infectious Diseases Society of America (IDSA), International Society for Anti-infective Pharmacology (ISAP), Society of Critical Care Medicine (SCCM), and Society of Infectious Diseases Pharmacists (SIDP). Pharmacotherapy.

[16] Bassetti M (2018). Rational approach in the management of *Pseudomonas aeruginosa* infections. Curr. Opin. Infect. Dis..

[17] Montravers P, Bassetti M (2018). The ideal patient profile for new beta-lactam/beta-lactamase inhibitors. Curr. Opin. Infect. Dis..

[18] Kollef MH (2019). Ceftolozane-tazobactam versus meropenem for treatment of nosocomial pneumonia (ASPECT-NP): A randomised, controlled, double-blind, phase 3, non-inferiority trial. Lancet Infect. Dis..

[19] European Medicines Agency (2019). Zerbaxa.

[20] Bassetti M (2019). Ceftolozane/tazobactam for the treatment of serious *Pseudomonas aeruginosa* infections: A multicentre nationwide clinical experience. Int. J. Antimicrob. Agents.

[21] Escolà-Vergé L (2018). Ceftolozane/tazobactam for the treatment of XDR *Pseudomonas aeruginosa* infections. Infections..

[22] Gallagher JC (2018). Ceftolozane-Tazobactam for the treatment of multidrug-resistant *Pseudomonas aeruginosa* infections: A multicenter study. Open Forum Infect. Dis..

[23] Haidar G (2017). Ceftolozane-tazobactam for the treatment of multidrug-resistant *Pseudomonas aeruginosa* infections: Clinical effectiveness and evolution of resistance. Clin. Infect. Dis..

[24] European Centre for Disease Prevention and Control (2015). European Surveillance of Healthcare-Associated Infections in Intensive Care Units—HAI-Net ICU Protocol, Version 1.02.

[25] Khwaja A (2012). KDIGO Clinical practice guidelines for acute kidney injury. Nephron. Clin. Pract..

[26] Knaus WA, Draper EA, Wagner DP, Zimmerman JE (1984). APACHE II: A severity of disease classification system. Crit. Care Med..

[27] Vincent JL (1996). The SOFA (Sepsis-related Organ Failure Assessment) score to describe organ dysfunction/failure. On behalf of the Working Group on Sepsis-Related Problems of the European Society of Intensive Care Medicine. Intens. Care Med..

[28] Charlson ME, Pompei P, Ales KL, MacKenzie CR (1987). A new method of classifying prognostic comorbidity in longitudinal studies: Development and validation. J. Chronic Dis..

[29] Edwards IR, Aronson JK (2000). Adverse drug reactions: Definitions, diagnosis, and management. Lancet.

[30] Pogue JM (2020). Ceftolozane/tazobactam vs polymyxin or aminoglycoside-based regimens for the treatment of drug-resistant *Pseudomonas aeruginosa*. Clin. Infect. Dis..

[31] Paterson DL, Isler B, Stewart A (2020). New treatment options for multiresistant gram negatives. Curr. Opin. Infect. Dis..

[32] Zaragoza R (2020). Update of the treatment of nococomial pneumonia in the ICU. Crit. Care.

[33] Gatti M, Giannella M, Raschi E, Viale P, De Ponti F (2021). Ceftolozane/tazobactam exposure in critically ill patients undergoing continous renal replacement therapy: A PK/PD approach to tailor dosing. J. Antimicrob. Chemother..

[34] Falagas ME (2010). Colistin therapy for microbiologically documented multidrug-resistant Gram-negative bacterial infections: A retrospective cohort study of 258 patients. Int. J. Antimicrob Agents.

[35] Papazian L, Klompas M, Luyt C-E (2020). Ventilator-associated pneumonia in adults: A narrative review. Intens. Care Med..

[36] Melsen WG (2013). Attributable mortality of ventilator-associated pneumonia: A meta-analysis of individual patient data from randomised prevention studies. Lancet Infect. Dis..

[37] European Medicines Agency (2020). Zavicefta.

[38] Sader HS, Carvalhaes CG, Shortridge D, Castanheira MC (2020). Comparative activity of newer β-lactam/β-lactamase inhibitor combinations against Pseudomonas aeruginosa from patients hospitalized with pneumonia in European medical centers in 2020. Eur. J. Clin. Microbiol. Infect. Dis..

[39] Jorgensen SCJ (2019). Real-world experience with ceftazidime-avibactam for multidrug-resistant Gram-negative bacterial infections. Open Forum Infect. Dis..

[40] Klompas M (2019). Ventilator-associated events: What they are and what they are not. Respir. Care.

